# Three-Dimensional Dosimetry by Optical-CT and Radiochromic Gel Dosimeter of a Multiple Isocenter Craniospinal Radiation Therapy Procedure

**DOI:** 10.3390/gels8090582

**Published:** 2022-09-13

**Authors:** Matheus Antonio da Silveira, Juliana Fernandes Pavoni, Alexandre Colello Bruno, Gustavo Viani Arruda, Oswaldo Baffa

**Affiliations:** 1 Departamento de Física, FFCLRP—Universidade de São Paulo, Ribeirão Preto 14040-901, Brazil; 2 Hospital das Clínicas da Faculdade de Medicina de Ribeirão Preto–USP, Ribeirão Preto 14015-010, Brazil

**Keywords:** 3D gel dosimetry, craniospinal irradiation, gel dosimetry, optical computed tomography, fricke-xylenol-orange dosimetry, intensity modulated radiation therapy (IMRT)

## Abstract

Craniospinal irradiation (CSI) is a complex radiation technique employed to treat patients with primitive neuroectodermal tumors such as medulloblastoma or germinative brain tumors with the risk of leptomeningeal spread. In adults, this technique poses a technically challenging planning process because of the complex shape and length of the target volume. Thus, it requires multiple fields and different isocenters to guarantee the primary-tumor dose delivery. Recently, some authors have proposed the use IMRT technique for this planning with the possibility of overlapping adjacent fields. The high-dose delivery complexity demands three-dimensional dosimetry (3DD) to verify this irradiation procedure and motivated this study. We used an optical CT and a radiochromic Fricke-xylenol-orange gel with the addition of formaldehyde (FXO-f) to evaluate the doses delivered at the field junction region of this treatment. We found 96.91% as the mean passing rate using the gamma analysis with 3%/2 mm criteria at the junction region. However, the concentration of fail points in a determined region called attention to this evaluation, indicating the advantages of employing a 3DD technique in complex dose-distribution verifications.

## 1. Introduction

Craniospinal irradiation (CSI) is a complex radiation technique employed to treat patients with primitive neuroectodermal tumors such as medulloblastoma or germinative brain tumors with the risk of leptomeningeal spread [1]. In adults, this technique poses a technically challenging planning process because of the complex shape and length of the target volume. Traditional CSI usually treats the entire central nervous system (CNS) with classic 3D conformal radiation therapy (3DCRT), which uses opposite lateral fields, including the brain and posterior fields, to treat the spine. Although effective in controlling the disease, 3DCRT does not spare any organs and causes significant acute and late morbidities depending on the dose delivered. In an attempt to maintain tumor control, improve dose conformity, and decrease dose to organs at risk (OAR), intensity-modulated radiation therapy (IMRT) and volumetric-modulated arc therapy (VMAT) have been used in clinical practice for CSI [2,3,4,5,6]. 

IMRT technology can offer a better conformity and homogeneity index than traditional multi-field 3DCRT [7,8]. Nevertheless, IMRT implementation for CSI treatment still requires strategies for matching the irradiation treatment fields with non-uniform fluency. The dose distribution in the junction region is critical to ensure that no hot spots could damage the patient’s spinal cord. Recently, some authors have proposed a new IMRT technique with the possibility of overlapping adjacent fields or junctions. Cao et al. proposed the jagged-junction IMRT in which a three-isocenter IMRT plan is used to address the junction issues by intentionally overlapping adjacent fields [4]. Afterward, Wang et al. developed the three-isocenter overlap-junction (TIOJ) IMRT, a simplified approach that reduced the planning and treatment time involved in using a larger beam overlapping region [5]. This new IMRT method makes it easier to control the target’s uniformity and reduces the number of hot spots in the junction area. Although it works well in the planning treatment volume, there is the potential for hot spots outside the target, especially in the regions where there is overlap from beam divergence, which can be challenging to detect in the quality assurance (QA). Thus, a rigorous QA check is essential to guarantee the real benefits of these novel techniques in clinical practice. Due to the dose distribution complexity, especially in the matching field regions, three-dimensional dosimetry (3DD) may be the ideal tool for QA. 3DD by gels, combined with an imaging technique, stands out for the absorbed-dose determination along the total irradiated volume, really simulating the treatment while giving information about the dose distribution in 3D [9,10,11].

Fricke gels were the first three-dimensional dosimeters proposed [12]. Their response to irradiation is based on the oxidation of ferrous ions (Fe^2+^) to ferric ions (Fe^3+^). However, the diffusion of the ferric ions blurred the spatial information. Various investigators proposed the addition of chelating agents to reduce this effect. Xylenol orange was one of these agents, which reduced the diffusion by chelating the ferric ions and produced a chemical species that has an intense absorption peak at 585 nm. Thus, the Fricke xylenol orange gel dosimeters change its color from yellow orange to deep purple upon irradiation [12], and, when combined with a three-dimensional imaging technique, demonstrates promising results in 3DD [13,14,15,16]. Recently, the addition of formaldehyde to the Fricke xylenol Orange (FXO-f) gel resulted in a higher melting point dosimeter and a similar dosimetric response as FXO [17]. Formaldehyde was used in other gel dosimeters to increase the melting temperature [18].

Optical computed tomography (OCT) is one of the emerging image techniques for performing 3DD in the clinical routine [19,20]. OCT determines, from the reconstruction of the acquired projections, the attenuation coefficient map related to the absorbed dose in a sample [20]. The standard reconstruction algorithm for X-ray CT reconstruction have been implemented in OCT dosimetry. For example, the FDK (Feldkamp, Davis, and Kress) algorithm [21], for reconstructions in cone-beam CT acquisition can be improved by the interactive reconstructions techniques, SIRT, SART, OSC [22,23] and combined to a variational method to suppress noise, for example, the total variation (TV) proposed by Rudin [24]. The published studies in reconstruction techniques allowed the determination of complex dose distributions [25,26].

For these reasons, this study applied FXO-f gel dosimeter and OCT for performing a 3D QA procedure in the matching field region of an IMRT CSI planning using multiple isocenters. As undertaken in the clinical routine, the plan was previously approved using a planar (two-dimensional) dosimetry with an ionization chamber array detector.

## 2. Results and Discussion

### 2.1. Calibration

**Figure 1:**
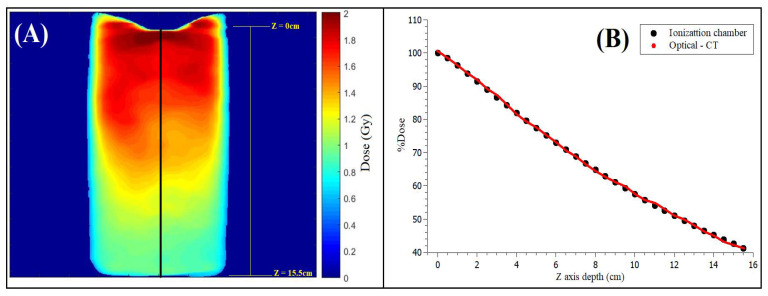
(**A**) Calibration curve achieved (black line) for calibration purposes from irradiation with 2 Gy delivered to the maximum dose depth. All the attenuation values outside the calibration range (0.8–2.0 Gy) were set to 0 Gy (dark blue). The black line represents the central axis used to build the PDD curve, using the inside gel values starting at a maximum 2 Gy dose value. (**B**) Measured PDD at the central axis of the gel phantom and the expected PDD value from the LINAC’s dosimetric table data.

The OCT image with attenuation coefficient contrast achieved from the PDD measurement (Figure 1A) allowed the PDD computation from the measured data. These values were compared to the PDD values achieved from the LINAC’s dosimetric table data (Figure 1B). 

**Figure 2:**
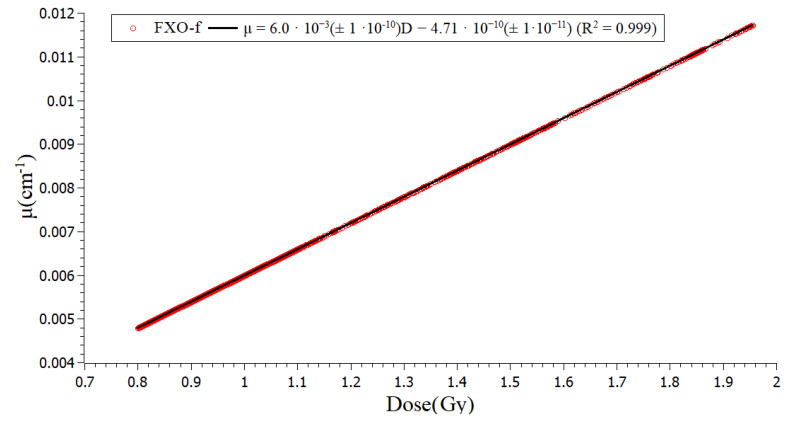
Calibration curve relating the optical attenuation coefficient (µ) achieved in the PDD OCT and absorbed dose delivered at the same point. A sensitivity of 6.0 × 10^−3^ cm^−1^/Gy was achieved. Calculated points are in red and linear fitting is the black line.

The calibration curve was built by relating the dosimetric table data PDD value, considering that the maximum dose delivered was 2 Gy, to the attenuation coefficient achieved in the same depth point (Figure 2). A linear relationship was achieved between the optical attenuation coefficient (µ) and the absorbed dose (D), with a sensitivity of 6.0·10^−3^ ± 1.0·10^−10^ cm^−1^/Gy and a correlation coefficient (R^2^) of 0.999. The linear behavior and the sensitivity value in the magnitude of 10^−3^ cm^−1^/Gy are in accordance with other papers in the literature [27,28]. This linearity justifies normalizing the dose distribution in the measured and calculated image for comparisons. The calibration curves, dose versus attenuation coefficient, starts at a dose of 0.8 Gy, corresponding to 40% of the total dose. 

A dose resolution of 0.1 Gy was achieved for this gel batch [29]. This calculation was based on the relation between the attenuation coefficient and absorbed dose achieved in the calibration curve [29]. 

### 2.2. 3D QA for CSI Treatment

**Figure 3:**
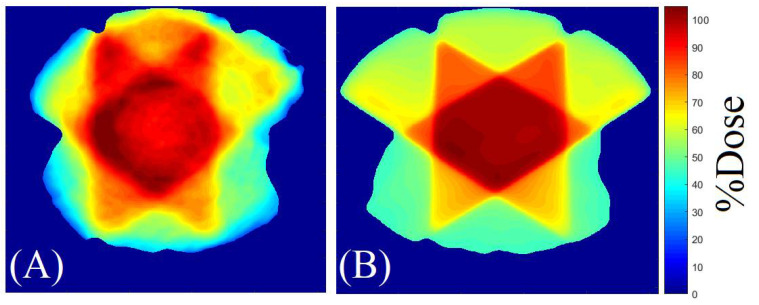
Dose distribution measured by the gel dosimeter with OCT scanning (**A**), and the corresponding slice (6.3 cm) calculated by the TPS (**B**)—20% of threshold.

**Figure 4:**
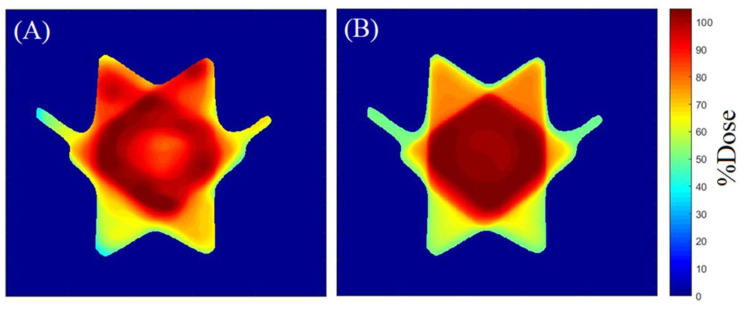
Dose distribution measured by the gel dosimeter with OCT scanning (**A**), and the corresponding slice (5.4 cm) calculated by the TPS (**B**)—40% of threshold.

**Figure 5:**
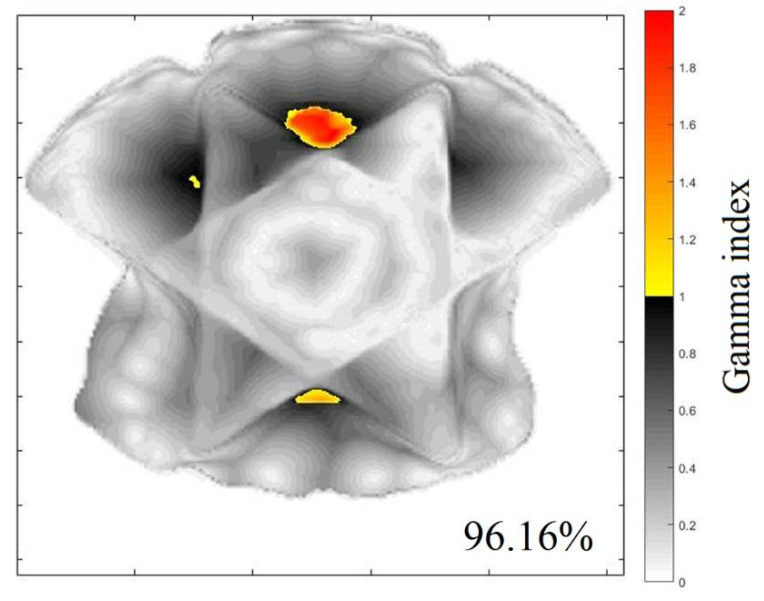
Gamma map index comparing the measured image and calculated image of Figure 8, with 95.57% of approved pixels (z = 6.3 cm) 20% of threshold.

**Figure 6:**
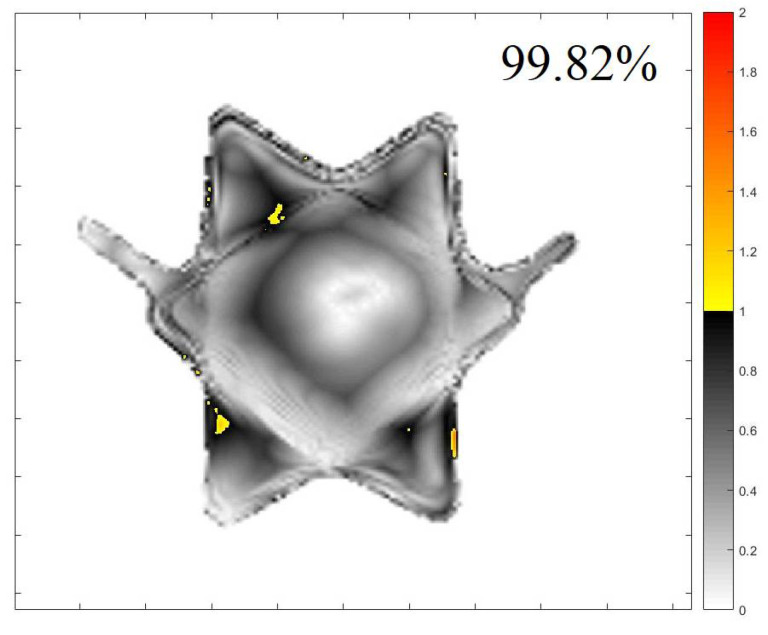
Gamma map index comparing the measured image and calculated image of Figure 9, with 99.82% of approved pixels (z = 5.4 cm) 40% of threshold.

Figure 3 and Figure 4 illustrate an example of the dose distribution at the junction region of the matching field achieved with the gel and expected by the TPS with the 20, and 40% thresholds applied, respectively. From these images, the similarity between the planned dose and the measured dose can be observed. The gamma map in Figure 5 and Figure 6 is an example slice of the gamma evaluation using 3%/2 mm/20%threshold criteria.

Table 1 shows the percentage of approved points in the gamma analyses at all slices evaluated at the junction region for both thresholds employed. The average approved pixels’ percentage in this measurement is 94.09% (86.24–96.16%) with 20% of the dose threshold and 94.05% (94.5–99.9%) with 40% of the dose threshold. These measurements are approved considering the established criteria. 

**Figure 7:**
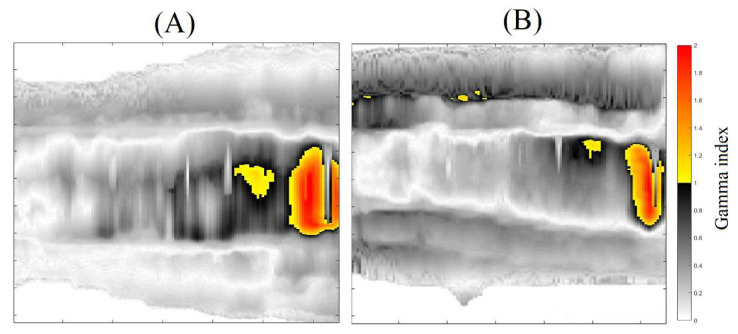
Gamma map images for the sagittal (**A**), and Coronal plans (**B**)—Threshold of 20%.

**Figure 8:**
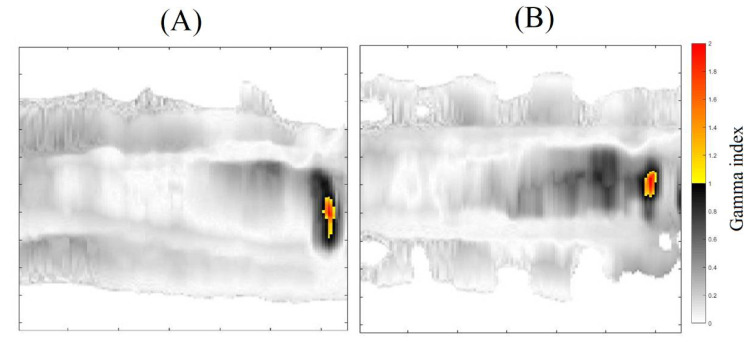
Gamma map images for the sagittal (**A**), and Coronal plans (**B**)—Threshold of 40%.

The 3DD approval importance is noteworthy because all the dose points in the matching field region were evaluated, which is different than in any other dosimetric technique. However, regions with less than 90% of approved pixels were found and called to attention; these include z coordinates between 7.8, 7.9, and 8.0 cm for the 20% threshold analysis and between 7.9 and 8.0 cm for the 40% threshold analysis. If we look at the sagittal and coronal views of the gamma analysis results, we can see the region where these fails are concentrated (Figure 7 and Figure 8 for the 20 and 40% of dose threshold, respectively). For both dose thresholds, the fails are almost in the same place with slight differences in size. In this case, even with the plan approval, the concentration of the fails in a delimitated region call attention and probably would deserve more investigations before the plan delivery. This information is unavailable in the usual pre-treatment QA with bidimensional detectors, which evaluate both fields separately and indicate the plan approval of both fields in this case.

This study employed two different dose thresholds to evaluate the dose distributions comprehensively. The first threshold of 20% was employed to check all the valid measured dose distribution, excluding only low-dose points, in the border of the measurement where possible image artifacts occurred when scanning the vial with the OCT. This analysis was possible only due to an extrapolation of the calibration curve to smaller dose values out of the calibration verification range. This procedure is acceptable because the gel FXO-f gel dosimetry linearity was already verified in the smaller dose values [17]. The second threshold of 40% evaluated only the dose interval in the calibration range at the expense of evaluating a smaller dose volume. As stated before, the results are similar, indicating approximately the main fail region around the same position.

No other 3DD measurement was reported in the literature for this irradiation type. Wang found a range of 92.5 to 97.5% with a mean of 94.5% of the approved pixel in 3%/2 mm gamma analysis using the 2D film dosimetry [1]. Zhou et al. used the ArcCHECK and EBT3 film dosimeters to perform CSI plan QA, analyzing the impact of setup errors. They presented the pass rate ranging from 85% to 95% on the gamma analysis with 3% dose deviation and multiples distance to agreement (1 mm to 8 mm) criteria [30]. Nguyen et al. used an electronic portal-imaging device panel as the detector and EPIDQA software with gamma analysis 3%/3 mm and accuracy above 90% as the pass criteria. They analyzed the two isocenters separately and found passing rates ranging from 94.5 to 99.9% in a threshold of 40% [31]. Therefore, the results found in the literature and in this paper are related and consistent for verifying a CSI plan using multiple isocenters. All of them verified the plan approval, but only the 3DD called attention to a specific failure region due to higher data provided for evaluation. However, it must be remembered that different treatment plans were evaluated in each study and that the intensity modulation level, which is related to the gamma pass rate, may vary among them. Finally, the complexity of this technique undeniably requires a more rigorous planning QA, such as the one provided by a truly 3D gel dosimeter.

## 3. Conclusions

These results show that gel dosimetry using the radiochromic gel FXO-f combined with OCT allows three-dimensional dose determination for a CSI procedure with complex multiple-field planning. The treatment planning evaluated was approved in our gamma analysis with 3%2 mm criteria, but a region with approval between 90 and 95% of approval appeared and deserved attention. Conventional QA cannot detect this information, revealing the importance of a true 3D quality assurance in complex clinical procedures.

## 4. Materials and Methods

### 4.1. FXO-f Gel Dosimeter Preparation

**Figure 9:**
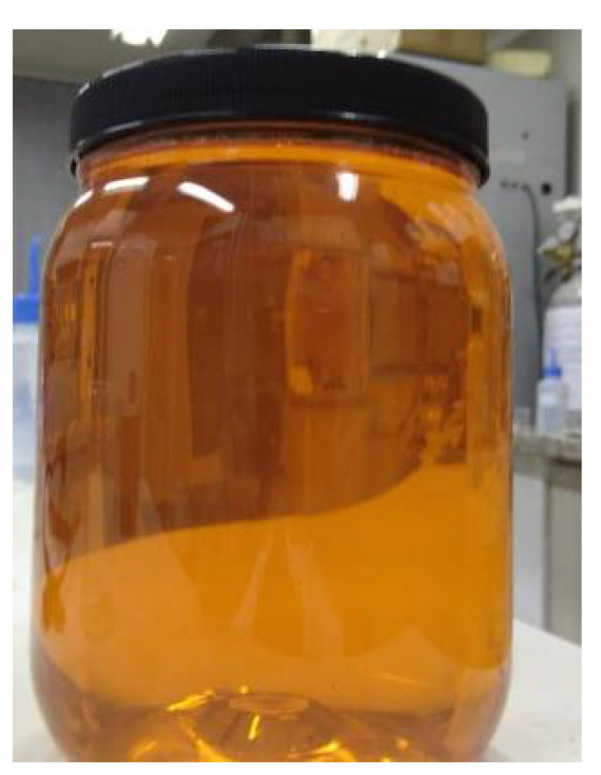
Cylindrical vial (20 cm tall and 15 cm in diameter) filled with FXO-f to perform 3D dosimetry.

FXO-f is a modified version of the Fricke xylenol orange gel dosimeter by adding formaldehyde. Its preparation starts with the dissolution of 8% gelatin (Bovine skin, 300 Bloom, Sigma-Aldrich, San Louis, MO, USA) in deionized water and then warming it up to 50 °C. Zinc pellets were added to the gelatin solution for 15 min to clean undesired peroxides and avoid the fast darkening of the gel. The solution was cooled down to 35 °C, and the 3% formaldehyde (Dinamica, Indaiatuba-SP, Brazil) was added. After five minutes of homogenization, 0.05 mM of xylenol orange (Sigma-Aldrich, San Louis, MO, USA) and 0.3 mM of ferrous ammonium sulfate (Sigma-Aldrich, San Louis, MO, USA) diluted in sulphuric acid (Dinamica, Indaiatuba-SP, Brazil) were added to the gelatin solution. The concentration of sulphuric acid in the final gel is 50 mM. Two cylindrical transparent plastic recipients, used in packing consumer products, were used to store the gel. These vials have a concave bottom, which is not ideal; however, they are readily available, cheap, and disposable. Recipients were 20 cm tall and 15 cm in diameter (Figure 9) and were filled with a total gel volume of 2.4 L. One was used for calibration in the percentage depth dose (PDD) curve measurement, and the other for the CSI matching field verification. The vials were left in the refrigerator for 32 h to guarantee the complete gelling process. This temperature also keeps the dosimeter color more stable, avoiding the auto-oxidation, consequently, the darkening over time [32].

### 4.2. CSI Treatment and Verification Plan Creation

Before the irradiations, an X-ray computed tomography (CT) of a similar cylindrical vial was acquired using a standard head and spinal protocol in the Philips Brilliance Big Bore scanner (Phillips Medical Systems, Cleveland, OH, USA)). This vial was filled only with gelatin (bovine gelatin bloom 250—Gelita^®^) with a concentration of 8 vol%, and the acquired images had 512 × 512 pixels resolution with a 0.5 × 0.5 mm^2^ effective pixel size. A reference point in the cylinder vial was marked, and a small piece of solder wire with a 3 mm diameter from Best (Allent Brasil soldas Ltd. a, Manaus, AM, Brazil) was imaged on it and worked as a radiopaque marker of the same reference point in the 3D treatment-planning software (TPS) and in the OCT images. 

The X-ray CT images of the cylindrical vial were imported to the Eclipse TPS, version 15.6 (Varian Medical Systems, Palo Alto, CA, USA) and were used as the base for the CSI verification plan creation. The dose calculation algorithm used was the analytical anisotropic algorithm (AAA), version 13.6.23, with a 2.5 mm calculation grid. A CSI plan using the simplified approach described in the TIOJ IMRT treatment protocol with two isocenters, one in the cranial region and the other in the spinal region [1], was selected, and the matching of these fields was verified. This plan delivered a total dose of 36 Gy (20 fractions of 1.8 Gy) with 6 MV intensity-modulated beams and 500 MU/min dose rate. 

**Figure 10:**
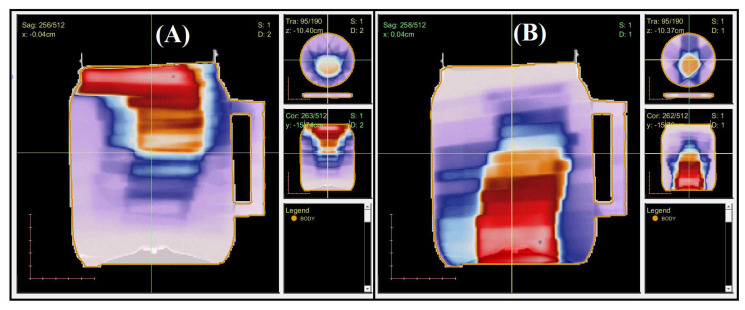
Three plan views of the planned dose distribution extracted from the TPS and visualized in CERR. (**A**)–Cranial IMRT field. (**B**)–Spinal IMRT field.

Therefore, it was necessary to create two verification plans, one for each field used. This step was undertaken because of our interest in evaluating the combined dose distribution at both fields junction. Figure 10 shows the dose distribution of each field of the CSI plan, recalculated in the cylindrical vial geometry in the verifications plans visualized using the CERR interface (Computational environment for radiotherapy research [33].

**Figure 11:**
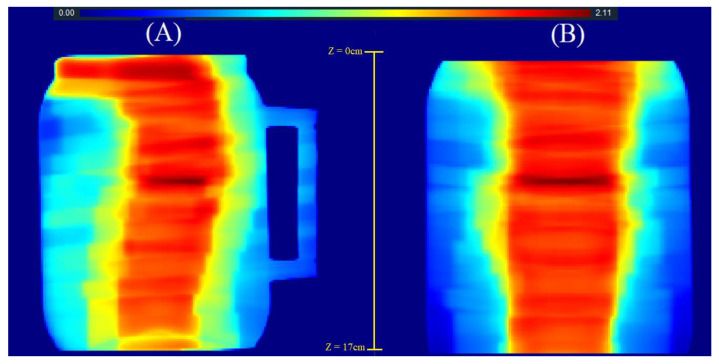
Complete dose distribution at the junction region of the treatment planning in the cylindrical vial geometry obtained by post-processing at Matlab^®^ 2016 with a different color map than CERR. The upper scale shows the dose range from 0 to 2.11 Gy. (**A**) Sagittal plane view. (**B**) Coronal plane view. An indication of the z-coordinate orientation of the slices is also presented.

The complete 3D dose distribution to be verified in the matching region was achieved by summing the presented dose-distribution data in 3D, using an in-house developed Matlab^®^2016 script (Mathworks Inc., Nattick, MA, USA). The complete junction dose distribution in the coronal plane is presented in Figure 11.

### 4.3. Irradiations

The irradiations were performed on a 6 MV Unique LINAC (Varian Medical Systems, Palo Alto, CA, USA).

**Figure 12:**
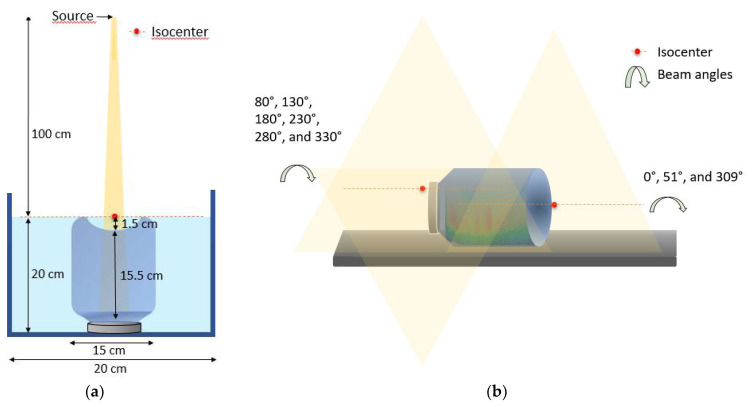
Irradiations setup for calibration (**a**) and for CSI dose verification (**b**).

**Figure 13:**
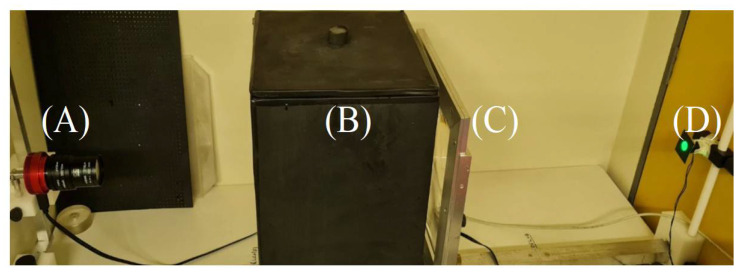
(**A**) ZWO ASI120 mm-S camera; (**B**) Water tank; (**C**) Fresnel lens and (**D**) 3 W LED.

All 3DD procedures using radiochemical gels require a calibration process for each gel batch prepared. The reason is that slight differences in the manufacturing process and/or the aging of the chemicals may result in different optical attenuation coefficients related to the absorbed dose [34]. In this experiment, the gel dosimeter calibration was undertaken using a PDD measurement (Figure 12a). One of the cylinders gel vials (15 cm diameter) was positioned upside down and inside a larger cylinder phantom (20 cm diameter) filled with water until the top of the gel vial. The larger phantom ensured at least 5 cm of gel and water beyond the field size borders to guarantee all the scattering necessary for the PDD measurement at the central axis [35]. As the gel vial was positioned upside down, its concave bottom had to be filled with water to allow the PDD measurement in the center of the vial with 100 cm of the source to surface distance (SSD) positioned in the water. The PDD curve achieved with irradiation of a 5 × 5 cm^2^ field size delivering 2 Gy at the depth of maximum dose with a dose rate of 500 MU/min was measured. The optical attenuation coefficients measured in the irradiated gel were related to the dose values received at each point to achieve the calibration curve. The dose values were extracted from LINAC’s dosimetric table data and are presented in Figure 13A. The institution’s quality assurance program periodically verifies these values, guaranteeing them. 

The gel vial irradiation for the CSI matching field verification followed the CSI plan. 

Considering that our phantom did not cover all the treatment region, it was necessary to locate both treatment isocenters projections in the LINAC couch to carefully position the gel phantom in the matching field region (Figure 12b). All the displacements necessary for the positioning and irradiation were based on the couch coordinates following the TPS values. The cylinder vial was first irradiated with the CSI cranial beam, and the spinal treatment field was delivered after the displacement. This way, the total treatment dose distribution in the matching field region was delivered to the same cylindrical vial. This gel vial was scanned by the OCT, and the measured dose distribution was compared to the TPS calculated dose distribution.

### 4.4. Scanning and Image Processing

#### 4.4.1. OCT Settings

The OCT used to scan the gel dosimeters was developed by our research group. It uses a cone beam geometry and is equipped with a ZWO ASI120mm-S, a high-resolution astronomic camera integrating a AR0130CS 1/3″ sensor, and 7–70 mm varifocal lens. Its water tank is filled with water and 8% sucrose (Cristal Sugar Patéko^®^) to match the light refraction index of the gel and its surroundings, avoiding undesired light refraction and the interfaces. The convergent light source is achieved using a point 3W LED coupled with a 545 nm bandpass filter and a Fresnel lens (focal distance of 25 cm). Figure 13 shows the optical-CT presenting the main components.

#### 4.4.2. Image Acquisition Protocol

To reconstruct a 3D attenuation coefficient map, the phantom was first scanned without any delivered dose before its irradiation (pre-scan data, *I*_0_). After the irradiation, the phantom was scanned again with the same protocol (scan data, *I*). The scannings were performed with a complete 360° rotation in 0.5° steps, resulting in the acquisition of 720 projections from the cylindrical phantom. Each projection was acquired with 800 × 800 pixels with an effective pixel size of 0.32 × 0.32 mm^2^. The primary reconstruction of the attenuation coefficient map in each pixel was undertaken with the FDK algorithm [21] for cone beam geometry, implemented in Matlab^®^2016 (Mathworks Inc. Nattick, MA, USA) to solve the line integral presented in Equation (1), for all the length L of the scanned gel volume.

(1) $$\int_{0}^{L}\mu \left(x\right)dx=-\ln\left(\frac{I}{I_{0}}\right)$$

However, to improve resolution and suppress noise, the simultaneous iterative algorithm (SIRT) combined with total variation minimization (TV) [22,23,24] was implemented, resulting in a high-quality image to compare with TPS. The total time of each scanning was 1.20 min, and the reconstruction time for SIRT-TV was 5 min. The final images were reconstructed with the same effective pixel size of 0.32 × 0.32 mm^2^ and 512 × 512 pixels.

#### 4.4.3. Image Comparisons

The fiducial marks were used to register the slices in the TPS with the OCT reconstructed images. In the clinical context, the images are compared using the gamma analysis, an index that indicates on a point-by-point basis if the compared dose distributions are similar considering a distance to agreement (DTA) and the percentage dose difference criteria [36]. We used a three-dimensional gamma-index analysis in a code implemented by our group in Matlab^®^2016 (Mathworks Inc., Nattick, MA, USA). All the dose volumes were compared with a search for similar dose points not only in the same image slice, but also in the previous or aftward image slices. Criteria of 3% dose difference and 2 mm of DTA [37] with a threshold of 20% to avoid edges scattering contamination and distortions and another result using a 40% within the calibration curve interval were employed. A gamma image of each dose slice was generated. The approval criteria used was that more than 90% of the pixels in the gamma image should be approved to validate the correct matching fields region.

## Figures and Tables

**Table 1:** Gamma’s pass rate for all slices in the junction region in the cylindrical vial. Cranial (Z = 4 cm) to spinal (Z = 8.1 cm) region.

Z (cm)	Gamma 20% (%)	Gamma 40 (%)	Z (cm)	Gamma 20% (%)	Gamma 40% (%)
4.0	92.76	99.86	6.1	96.02	99.76
4.1	92.32	99.88	6.2	96.15	99.86
4.2	92.46	99.96	6.3	96.16	99.84
4.3	93.21	99.96	6.4	95.72	99.76
4.4	92.51	99.93	6.5	95.12	99.77
4.5	92.32	99.95	6.6	94.88	99.71
4.6	92.40	99.87	6.7	94.84	93.95
4.7	92.86	99.76	6.8	94.79	94.15
4.8	93.33	99.97	6.9	94.28	94.24
4.9	93.30	99.91	7.0	95.26	94.00
5.0	93.28	99.90	7.1	95.40	93.67
5.1	93.29	99.88	7.2	94.87	93.38
5.2	95.01	99.85	7.3	95.77	93.07
5.3	95.42	99.84	7.4	96.06	92.84
5.4	95.57	99.82	7.5	95.88	92.60
5.5	95.80	99.85	7.6	94.82	92.45
5.6	95.92	99.91	7.7	93.49	92.24
5.7	95.52	99.76	7.8	86.24	92.01
5.8	94.87	99.95	7.9	87.35	91.49
5.9	94.52	99.97	8.0	88.76	91.03
6.0	94.35	99.80	8.1	94.33	96.70

## Data Availability

Not applicable.
